# Dynamic Psychotherapy as a PTSD Treatment for Firefighters: A Case Study

**DOI:** 10.3390/healthcare10030530

**Published:** 2022-03-14

**Authors:** Joana Proença Becker, Rui Paixão, Manuel João Quartilho

**Affiliations:** 1 Faculty of Psychology and Educational Sciences, University of Coimbra, 3000-115 Coimbra, Portugal; rpaixao@fpce.uc.pt; 2 Faculty of Medicine, University of Coimbra, 3000-548 Coimbra, Portugal; mjquarti@ci.uc.pt

**Keywords:** dynamic psychotherapy, post-traumatic stress, case study, firefighters

## Abstract

In Portugal, forest fires are responsible for disasters that tend to be repeated annually, leading to dramatic consequences, such as those that have occurred in 2017, with the destruction of hundreds of houses and the deaths of dozens of people. Firefighters who are exposed to these potentially traumatic events are considered a high-risk group for the development of stress-related disorders. The aim of this study was to monitor the progress of two firefighters with symptoms of post-traumatic stress disorder (PTSD) treated through dynamic psychotherapy (DP) and to assess the feasibility of implementing this intervention within fire departments. A female firefighter and a male firefighter, with similar sociodemographic characteristics and PTSD symptom severity, were selected to verify the treatment applicability for both genders. The symptomatology changes were assessed through a set of instruments (PHQ-15, PCL-5, BSI, DASS, and CALPAS-P) applied every three months over 15 months (including pre-treatment, treatment period, and post-treatment). DP seemed to be an effective treatment for PTSD symptoms, with patients showing a state of increasing improvement even after the end of treatment. The acceptability to firefighters, the treatment adherence, the therapeutic alliance, and the reduction in PTSD symptoms suggest feasibility for implementing this intervention inside the Portuguese fire departments.

## 1. Introduction

Forest fires hit Portugal every year, leading to dramatic consequences such as those that occurred in 2017, with the destruction of hundreds of houses and the deaths of dozens of people. In that case, as in many other potentially traumatic events, the attention of media, government, and health professionals is often to primary victims. In contrast with primary trauma, which concerns the direct victim of an event, secondary post-traumatic stress is a consequence of knowing about a traumatic event experienced by another person, attempting to help others, or even listening to reports of suffering, as is the case of professionals who work in catastrophe scenarios [1]. Fortunately, a movement to raise awareness about the health of rescue workers (firefighters, emergency medical responders, police officers, etc.) was carried out by those who are concerned with the widespread psychological consequences of catastrophes [2].

Despite the inconsistency in the rates of prevalence of PTSD among professionals—namely, firefighters [1,3,4,5]—a set of factors has been associated with stress-related diseases in these professionals. Studies [6,7] have shown that the establishment of trauma exceeds exposure to the potentially traumatic event, being crossed by occupational and personal characteristics, interpersonal relationship, and physical and mental health conditions. Professionals who work in catastrophe scenarios may experience somatic symptoms, fatigue, PTSD, depression, anxiety, and alcohol abuse. According to Fraess-Phillips, Wagner, and Harris [8] (p. 21), there is a consensus in the literature that “firefighters are at a greater risk of experiencing an adverse stress reaction such as PTSD”. In contrast, some studies have suggested that firefighters, due to their training and experience, are more protected from being affected by a stress reaction than the general population [7,9]. However, cultural issues and the fear of stigma have been presented as the reasons for low rates of PTSD in firefighters [6,10,11].

The psychological impact of extreme events has motivated the implementation of evidence-based strategies and interventions aimed at survivors, including professionals who work in catastrophe scenarios. Psychological first aid has been considered a useful strategy in the immediate aftermath of disasters and terrorism as it “is designed to reduce the initial distress caused by traumatic events and to foster short- and long-term adaptative functioning and coping” [12] (p. 5). Its basic objectives are: enhance safety; provide physical and emotional comfort; identify and offer practical assistance to help affected people address their immediate needs and concerns; support adaptive coping; encourage affected people to take an active role in their recovery; provide information about self-care and mental health services. Psychological first aid precedes a psychological intervention; therefore, the professionals who deliver it must be attentive to the reactions of the victims and must have knowledge about the services offered in the affected areas. Contrary to some studies, evidence-based strategies for psychological first aid advise against debriefing [12].

Although debriefing has been pointed out as a tool for dealing with traumatic experiences, even being associated with post-traumatic growth [13], evidence has suggested that debriefing may not be a proper solution for overcoming traumatic experiences, since it is important to respect individuals’ adaption process, as well as “individual differences in premorbid vulnerability, distress severity, or social context” [14] (p. 127). In the same sense, peer support programs have been developed, “with trained peer supporters offering low-level psychological intervention, identifying at-risk colleagues, and facilitating pathways to professional help” [15] (p. 594). The results of these programs remain questionable, as there is not enough evidence on their effects [14]. Regardless of the controversies surrounding the means for preventing or minimizing the development of stress reactions among firefighters, it also seems necessary to investigate effective interventions for when symptoms have emerged.

International clinical practice guidelines (Phoenix Australia, NICE, APA) have strongly recommended trauma-focused therapies in the treatment of PTSD symptoms—namely, cognitive processing therapy (CPT), trauma-focused cognitive behavioral therapy (TF-CBT), and prolonged exposure (PE). Eye movement desensitization and reprocessing (EMDR) has also been strong recommended by these guidelines, with the exception of the APA guideline, which gave EMDR a moderate rating [16]. Regarding psychodynamic therapies, according to NICE guideline [17], evidence suggests significant benefits on PTSD remission. However, the evidence is insufficient, and therefore, such interventions are not recommended.

Although trauma-focused therapies had been pointed out as the most effective interventions for PTSD symptoms, there is also evidence supporting the effect of non-trauma-focused therapies [18]. However, there is a lack of comparative studies on different treatments for different types of conditions and patients. “Despite the significant symptom improvement, most patients do not fully recover with the existing time-limited treatments. Many PTSD patients will need longer periods or episodes of treatment of different types to fully recover” [19] (p. 253).

Psychoanalytic theory has made it possible to think about factors behind manifested symptoms and behaviors [20,21], and although dynamic psychotherapy (DP) is not considered first-line treatment, it is “still a common treatment for PTSD” [22] (p. 1). DP “emerged of overcoming the limitations of traditional psychoanalysis, responding to a broader demand of patients who seek help to cope with specific problems in a short-term. Especially when symptoms provoke social and occupational impairments, patients need a short-term solution, making it essential to focus on symptoms and sufferings that led them to seek the treatment” [23] (p. 1). In DP, the patient “must reconcile the occurrence of the traumatic event and its meaning with his or her concept of the self and the world” [24] (p. 416), understanding “the effect of the traumatic event on his or her personality, embedding in the context of his or her current experience” [19] (p. 2). According to D’Andrea and Pole [25] (p. 444), the psychodynamic process “may be more effective for trauma survivors than commonly realized”, as the focus of the treatment is on raising awareness of unconscious thoughts and feelings associated with the traumatic event. Additionally, the authors emphasized that a goal of DP is to reconstruct memories and meanings that may slow down “automatic processing of trauma stimuli and enhanced emotion regulation” in the face of traumatic experiences.

Considering the abovementioned findings, the current study aimed to investigate the effects of DP in the treatment of firefighters who fought the 2017 forest fires in Portugal and had developed post-traumatic stress symptoms. In addition, this study verified the feasibility of implementing this intervention within fire departments. “Firefighters are used to dealing with physical discomfort, unpredictability, the pressure and expectation of society and their departments, the fear of the victims and their own fears. However, being prepared to face extreme situations is not synonymous with being immune to traumatic stress” [2] (p. 80).

## 2. Materials and Methods

This study followed the American Psychological Association (APA) guidelines [26,27], considering the assessment through a set of instruments applied to identify PTSD symptoms and stress-related disorders (depression, anxiety, sleep disturbance, and somatic symptoms) every three months in order to monitor the progress of the patients throughout 15 months (pre-treatment, treatment, and follow up). The results of the measurement instruments have served as indicators of the progress of the therapeutic processes, especially regarding symptoms reduction, while the qualitative analysis allowed us to verify the feasibility of implementing this intervention within fire departments.

When this study was conducted, seven firefighters had completed their treatments (5 male and 2 female). The sample selection criteria for this case study were: firefighters who fight forest fires, those with a high score on the PCL-5 scale in the first assessment, and whose symptoms were related to their duty. Thus, a female firefighter and a male firefighter with similar sociodemographic characteristics and stress-related symptoms were selected, which made it possible to verify the applicability of the treatment to both genders. The subjects gave their informed consent before they participated in the study. The study was conducted in accordance with the WMA International Code of Medical Ethics, and it was approved by the Ethics Committee of the Faculty of Psychology and Educational Sciences of the University of Coimbra.

### 2.1. Measures

The first assessment (pre-treatment) was composed of a clinical interview and a questionnaire that included questions on sociodemographic characteristics and stressful experiences, with the addition of a set of instruments that measured the presence and severity of psychopathological symptoms—instruments that were also applied in the subsequent assessments. The measurement instruments were:

The Patient Health Questionnaire-15 (PHQ-15), which [28] inquiries about “15 somatic symptom or symptom clusters that account for more than 90% of the physical complaints reported in the outpatient setting” [28] (p. 259). It asks respondents to indicate how much they have been bothered by symptoms within the past four weeks, and the response format is a 3-point Likert scale. Symptom severity is measured on a scale of 0–30: 0–4 = no minimal; 5–9 = low; 10–15 = medium; and 15–30 = high. The PHQ-15 has been widely used as a screening instrument for somatic symptom disorder in healthcare settings and scientific research, as it is considered reliable and valid for general and clinical populations (Cronbach’s alpha = 0.80) [28,29].

The PTSD CheckList-5 (PCL-5) [30] is a 20-item self-reported measure that corresponds to the 20 DSM-5 symptom criteria for PTSD. This instrument, which is a 5-point Likert scale, can be applied to monitor symptom change, screen individuals for PTSD, and make a provisional diagnosis. The PCL-5 can be scored in different ways: a total symptom severity score (range of 0–80) can be obtained by summing the scores for each of the 20 items; DSM-5 symptom cluster severity scores can be obtained by summing the scores for the items within a given cluster; or a provisional PTSD diagnosis can be made by treating each item rated as moderate or high as a symptom endorsed, then following the DSM-5 diagnostic criteria.

The Brief Symptom Inventory (BSI) [31] is an instrument that assesses the presence of 9 dimensions of symptoms: psychoticism, somatization, hostility, obsessions–compulsions, paranoid ideation, anxiety, phobic anxiety, depression, and interpersonal sensitivity. The reliability for these dimensions ranges from 0.62 to 0.80 (Cronbach’s alpha). This self-report questionnaire asks respondents to indicate how much they have been bothered by a set of symptoms and feelings over the past week, and the response format is a 5-point Likert scale [31,32].

The Depression Anxiety and Stress Scale (DASS-21) [33], which aims to measure the emotional state of depression, anxiety, and stress, asking respondents to indicate how much the statements apply to them over the past week. The response format of DASS-21 is a 4-point Likert scale [34]. Scores of each dimension are calculated by summing the corresponding items, and recommended cut-off scores are: Depression: 0–9 = normal, 10–13 = mild, 14–20 = moderate, 21–27 = severe, 28+ = extremely; Anxiety: 0–7 = normal, 8–9 = mild, 10–14 = moderate, 15–19 = severe, 20+ = extremely severe; Stress: 0–14 = normal, 15–18 = mild, 19–25 = moderate, 26–33 = severe, 34+ = extremely severe. The overall score, which includes all items, presents high consistency (Cronbach’s alpha = 0.88) [33].

In the assessments applied throughout the treatment period, the California Psychotherapy Alliance Scale—Patient Version (CALPAS-P) [35] was included. The 24 items are distributed in four subscales: Patient Commitment Assessment (CP); Patient Working Capacity Assessment (PWC): Therapist Understanding and Involvement Assessment (TUI); and Agreement on Objectives and Strategies (WSC). Each of these subscales is calculated by scoring items from 1 to 7. CALPAS-P “is influenced both by the traditional psychoanalytic perspective, on the alliance perspective, as by the pan-theoretical model of Bordin” [36] (p. 67).

### 2.2. Clinical Cases

#### 2.2.1. Case 1

Anna was a 37-year-old single woman with 13 years of schooling and had been a professional firefighter for 14 years. At the diagnosis interview, Anna reported having experienced traumatic events in her work as firefighter, especially related to the violent forest fires that hit Portugal in 2017. Consequently, she described a set of symptoms that met criteria for post-traumatic stress disorder—namely, avoidance behavior, irritability, sleep disturbance, persistent inability to experience positive emotions, and prolonged psychological distress upon exposure to cues that symbolized or resembled an aspect of the traumatic events. Regarding her physical health, Anna complained of gastrointestinal problems, fatigue, dizziness, and recurring stomachache, headache, back pain, and joint pain that could not be explained by a medical condition. At the first assessment, Anna met criteria for PTSD and somatic symptom disorder.

#### 2.2.2. Case 2

Bruno was a 34-year-old woodworker with 14 years of schooling and had been a volunteer firefighter for 16 years. At the diagnosis interview, Bruno referred to the rescue of victims of a bus accident as the first time he felt stress-related symptoms and sought professional help. According to Bruno, despite having questioned leaving the voluntary service, being with his comrades in the fire department helped him to overcome the symptoms. However, in 2017 Bruno faced extreme situations that brought the symptoms back even more strongly than at the time of the event of a decade ago. Bruno reported suffering with avoidance behavior, intrusive distressing memories, nightmares (in which the content is related to the 2017 forest fires), irritability, exaggerated startle response, and psychological reactions to reminders of the traumatic events (both the bus accident and the 2017 forest fires). Regarding his physical health, Bruno mentioned back and joint pains, fast heartbeat, gastrointestinal problems, poor sleep quality, and fatigue, but he had showed no concern and said that he did not devote energy to such symptoms. At the first assessment, Bruno just met DSM-5 [37] criteria for PTSD. 

### 2.3. Therapy

The therapist, who is the main author of this study, has more than 6 years of experience in DP and had the selected cases supervised by therapists with more than 20 years of experience. The treatments consisted of 50-min weekly sessions. 

The treatments followed the main steps well-described in the Psychodynamic Psychotherapy: A Clinical Manual [38], such as: (a) Establishing therapeutic alliance. (b) Listening to sounds (tone, volume, and timbre) and silence (the rhythm of sound stops and starts). “If you listen to silence, you begin to realize that it sounds different at different times” (p. 145). (c) Reflecting about sounds and silence in order to understand its meanings (patient’s affects, therapist’s feelings—countertransference). This phase is where therapist assesses therapeutic alliance, the phase of the treatment and use his or her clinical experience and knowledge of theory and technique to understand what is happening in patient’s mind and how much patients are able to listen to and use what therapist has to say. (d) And finally, intervening, which may be basic, supporting, or uncovering. “Basic interventions are to gather history, teach patients to use the treatment, and convey understanding”. Supporting interventions are to supply resources and assist patients in using their own resources. In addition, “uncovering interventions are to enhance the patient’s awareness of unconscious thoughts and feelings” (p. 158). Although patients were assessed every three months, the results were not communicated to them during the treatment. At the end of the treatment, the therapist and patients analyzed goals and the therapeutic processes, with a realistic appraisal of change.

### 2.4. Data Analysis

Treatment response was measure by examining symptoms reduction through the comparison of the assessment outcomes. The data analysis considered the pre-treatment as baseline, comparing its outcomes with the subsequent assessments in order to monitor the evolution of symptoms throughout the therapeutic processes. 

The feasibility of implementing the intervention inside fire departments was analyzed based on the following aspects: (a) receptivity of firefighters; (b) the treatment adherence; (c) therapeutic alliance; and (d) symptom reduction.

## 3. Results

Both patients met criteria for PTSD at the clinical interview that preceded their treatments. A PCL-5 cutoff score between 31 and 33 is indicative of probable PTSD [30], with Bruno scoring 43 and Anna 42. Although Bruno scored 16 and Anna 15 on the PHQ-15, which is indicative of high somatic symptom severity [28], Bruno did not meet criteria for somatic symptom disorder, as the symptoms did not result in significant disruption of his daily life, and he did not devote energy to these symptoms or health problems. On the other assessment measures (BSI and DASS-21), patients scored for depression and anxiety, as well as for the BSI dimensions hostility and paranoid ideation.

The aim of this study was to monitor the progress of PTSD patients treated with DP over a period of up to 12 months. However, the duration of the therapeutic processes was not specified. Bruno’s treatment lasted 40 weeks, while Anna needed 48 weeks to overcome her symptoms. Although Bruno had showed a significant reduction in the PCL-5 score in the third month of treatment, both patients scored for PTSD in the 6-month assessments, which may be related to apprehensions aroused by the critical fire period in Portugal. On the subsequent assessments, patients’ PCL-5 scores gradually decreased (Table 1). Bruno reached the treatment goal in the ninth month of treatment, having no more PTSD symptoms, although he still had scores that indicated the presence of psychopathological symptoms such as anxiety and depression (Table 2). Anna, on the other hand, needed a longer treatment, but had a progressive reduction in all symptoms over the 12 months of psychotherapy.

Both patients showed a significant improvement at the 9-month assessment (Table 2). However, Bruno’s subsequent assessments indicated symptoms of stress, anxiety, and depression, which may be explained by the fact that Bruno found out that his girlfriend was pregnant. As Bruno’s first follow-up assessment showed a worsening of psychopathological symptoms, a second follow-up assessment was performed after 3 months. In this one, the scores of all instruments were lower, in addition to there being an absence of PTSD symptoms.

The CALPAS-P scores (Table 3) showed that a good therapeutic alliance was established in both treatments, with higher scores in the dimensions WSC (4.6–7) and TUI (5–7). Anna had increasingly higher scores on the assessments, indicating greater working capacity and commitment to therapy and an increasingly positive evaluation of her therapist’s work. On the other hand, Bruno’s scores decreased from assessment 1 to the last assessment of the therapeutic alliance, completed in the last month of treatment.

Regarding the feasibility of implementing this intervention, the analysis of the four aspects (receptivity of firefighters; treatment adherence; therapeutic alliance; and symptom reduction) have shown that the Portuguese fire departments would benefit from this type of mental health service. The fire chiefs of the departments where the intervention was implemented provided the necessary structure for the therapist’s work—an isolated setting with suitable furniture in a building easily accessible by firefighters. In addition, fire chiefs and other high-ranking firefighters encouraged their squad to respond to the questionnaire and, if it was the case, initiate therapy. 

Firefighters’ receptiveness increased as they learned about the therapist’s work and the benefits reported by colleagues who were under therapy. Despite the recognition of the results obtained through DP in the treatment of PTSD, few firefighters sought treatment. Firefighters under treatment were committed to their therapeutic processes, rarely missing or delaying sessions. All those who started the treatment showed good adherence, with a rapid establishment of the therapeutic alliance, and had a significant improvement in their clinical conditions and in the way of dealing with adversities.

Fire chiefs of other departments had contacted the main researcher in order to request this intervention in their departments, which may indicate that the implementation of this intervention seems to be feasible in the Portuguese fire departments.

## 4. Discussion

Implementing a psychological intervention inside fire departments is challenging due to the reluctance of this population to admit emotional distress. The attitude of high-ranking firefighters, encouraging their squads to participate in the research and to seek therapy when they felt any symptoms of post-traumatic stress, was fundamental for the accomplishment of this study. Probably, the firefighters’ receptivity was influenced by the fact that this study was carried out after the violent forest fires of 2017, when the consequences of the catastrophe, including the victims’ mental health, were massively addressed by the media. However, although they highlighted the importance of this type of intervention, only a small group of firefighters sought treatment. The stigma related to mental disorders seems to have been the main reason for the low number of adherents.

Firefighters are considered a high-risk group for the development of stress-related disorders, as they are often exposed to extreme situations. The development of stress-related disorders is influenced by a set of factors such as personality traits, interpersonal relationships, working conditions, and mental and physical health issues [5,6,39,40]. These subjective and social factors interact with each other, affecting the perception of stress, which can be considered to be mainly responsible for stress in firefighters [2]. Considering the relevance of subjective appraisal, it seems to be reasonable to provide a treatment that is not focused on symptoms alone but on their meaning, which is the focus of psychodynamic models [2,41].

DP is a technique that considers the specificities of each patient, which precludes a detailed description of the therapeutic process. Nevertheless, it is worth noting that “DP has traditionally placed emphasis on insight into relationship patterns and processing avoids emotions, including unconscious thoughts and feelings associated with traumatic events” [41] (p. 101). The aim of this technique is to enhance self-understanding, which may allow patients not only to reach their goals but also to continue to improve after the end of treatment [23]. 

Anna and Bruno showed a significant improvement in PTSD symptoms throughout their therapeutic processes, a progress that remained evident at the post-treatment assessments. This finding corroborates clinical studies that “have shown that patients benefit from DP, and that psychoanalysis-based psychotherapies have presented a continuous state of improvement in patients” [41] (p. 93). Patients had presented different treatment times and oscillations in the severity of psychopathological symptoms, which can be explained by the fact that DP is conducted considering the patients’ capacity to process and tolerate feelings, which reflects directly on the length of therapy [23,41,42]. Since the focus of DP is on raising awareness of unconscious thoughts and feelings related to traumatic events, the patients needed time to slow down the automatic processing of trauma stimuli and enhance emotion regulation in facing their experiences [25].

In addition to symptom reduction, treatment adherence and the establishment of therapeutic alliance were other indicators of the feasibility of providing DP within fire departments. In the CALPAS-P, which evaluates the therapy features (from the patient perspective), both patients had high scores throughout their treatments, with higher scores in the dimensions that assess the therapist’s stance. Bruno had a decrease in the scores of all dimensions (even so, WSC and TUI were the highest), which seems to be related to the approach at the end of his treatment. Patients had been told that the project would last about a year and that its focus was on PTSD symptoms, which may have influenced the latter assessments. Finally, the recognition of the benefits obtained by firefighters treated through DP—consequently reflecting the work of the entire team and the interest of other departments in providing this intervention for their employees—leads to the belief that it is possible and necessary to offer mental health care tailored to this population.

This study has some limitations. First, as a case study, it is not possible to generalize the outcomes, mainly because it presents data from a small sample. Second, it is not a comparative study, so it is not possible to assume that DP is a more effective treatment than other psychological therapies. Third, a single-subject case experimental design was not employed to formally evaluate treatment effects—an approach that would be helpful to employ in future work. Despite these limitations, we believe that our study provides relevant information about an unexplored psychological intervention (DP), in addition to addressing a population that has been a target of PTSD research. Through the analysis of the feasibility of implementing DP within fire departments, we draw attention to the specificities of this population, while demonstrating its change in perspective and gradual acceptance of interventions aimed at mental health.

## 5. Conclusions

The effectiveness of DP remains questionable due to the scarcity of studies in this field. As a long-term treatment, DP is outside the front-line of treatments for mental disorders, as well as when pondering the implementation of mental health programs in institutions such as fire departments. “However, more than finding express solutions to their symptoms and conflicts, patients are interested in long-term results, which DP seems to achieve” [41] (p. 114). This is a preliminary study from longitudinal research. The comparison of DP outcomes in critical periods may be essential since the aim of DP is raising awareness of unconscious feelings and the reasons behind reactions and behaviors, enabling patients to face and deal with their life events.

## Figures and Tables

**Table 1:** PCL-5 scores.

Patients	Baseline	3 Months	6 Months	9 Months	12 Months	15 Months
Anna	42	39	39	30	6	5 *
Bruno	43	25	39	13	10 *	9 *

* follow-up assessments.

**Table 2:** Patients’ assessments.

Patients	Baseline	3 Months	6 Months	9 Months	12 Months	15 Months
Anna						*
PHQ-15	15	10	13	11	17	11
BSI GSI	2.39	1.81	2.19	1.02	0.89	0.60
BSI PST	50	49	49	23	23	27
BSI PSDI	2.54	1.95	2.35	2.34	2.04	1.18
DASS-21 stress	28	24	8	8	12	12
DASS-21 anxiety	16	12	12	2	0	4
DASS-21 depression	38	24	26	6	0	0
Bruno					*	*
PHQ-15	16	12	13	8	15	14
BSI GSI	1.75	1.91	2.21	0.66	1.75	1.66
BSI PST	48	47	48	35	49	52
BSI PSDI	1.91	2.14	2.43	1	1.89	1.69
DASS-21 stress	12	14	14	14	20	12
DASS-21 anxiety	14	16	12	8	20	14
DASS-21 depression	12	10	10	10	14	12

* follow-up assessments.

**Table 3:** CALPAS-P scores.

Patients	3 Months	6 Months	9 Months	12 Months
Anna				
PC	6.16	4.6	5.5	6.16
PWC	4	4	4.5	5
TUI	6.16	6.83	7	7
WSC	6.33	6.33	6.66	6.66
Bruno				
PC	5.33	5.83	4.5	
PWC	5	4	4.5	
TUI	6.66	6.16	5	
WSC	7	6.5	4.6	

## Data Availability

Not applicable.
